# Reproductive Immunology and Pregnancy

**DOI:** 10.3390/ijms23126485

**Published:** 2022-06-10

**Authors:** Dariusz Szukiewicz

**Affiliations:** Department of Biophysics, Physiology & Pathophysiology, Faculty of Health Sciences, Medical University of Warsaw, 02-004 Warsaw, Poland; dariusz.szukiewicz@wum.edu.pl

This Special Issue has been prepared to showcase the dynamic and comprehensive development of reproductive immunology, including the immunology of pregnancy. In a clinical context, the focus will be on the interactions between immune-system and reproductive-system functions at different stages, such as hormonal activity during the menstrual cycle; the production and transport of oocytes and sperm cells; the transport of dividing zygotes; implantation; intrauterine growth of the fetus and placenta; and the onset of labor. The constantly increasing pool of fertility disorders related to immune system dysfunction that lead to autoimmunity is an essential area of research [1]. In addition to the original research papers comprising the avant-garde in this field, comprehensive reviews provide information about the current state of the art. Both types of articles are included in this Special Issue.

Neonatal lupus erythematosus (NLE) refers to a clinical spectrum of symptoms (e.g., cardiac or cutaneous) and systemic abnormalities found in children whose mothers have produced autoantibodies against soluble antigens of the cell nucleus [2]. Among the autoantibodies of significant importance in NLE, anti-Ro (SS-A, Sjögren Syndrome type A), anti-La (SS-B, Sjögren Syndrome type B), and anti-ribonucleoprotein antibodies (anti-RNP) are listed most frequently in recent publications. The condition is rare, with a prevalence of 2% in mothers with anti-Ro and/or anti-La antibodies, and is usually benign and self-limited. However, the true prevalence of NLE may be underestimated due to the high proportion of unrecognized cases [3]. Moreover, passively acquired autoimmunization in NLE produces a multiform picture of the disease that may, sometimes, be associated with severe sequelae. Gryka-Marton et al., 2021 [4] reviewed, summarized, and updated the previous literature about NLE, focusing on the current state of knowledge about the role of anti-Ro in the inflammatory process.

Tumor necrosis factor-alpha (TNF-α) is a multifunctional type-1 helper (Th1) cell cytokine that plays essential roles in diverse cellular events, such as cell survival, proliferation, differentiation, and death [5]. This also applies to gestation, during which TNF-α plays beneficial roles in the development of early pregnancy by enhancing placental maturation and differentiation, embryonic development and parturition [6]. At the same time, however, the capacity for increased levels of TNF-α to be detrimental to pregnancy has been established beyond reasonable doubt. This dichotomy in TNF-α action during pregnancy may be related to the changed profile of this cytokine resulting from an abnormal balance between Th1 and Th2 cells. According to the immune maladaptation hypothesis, an abnormal cytokine profile, including elevated TNF-α, arises from the inappropriate regulation of normally Th2-deviated maternal immune responses; this leads to a shift toward harmful Th1 immunity [7]. Most importantly, TNF-α plays a central role in the pathogenesis of several inflammatory conditions. Consequently, anti-TNF-α drugs have been developed. Tumor necrosis factor (TNF)-alpha inhibitors, including etanercept (E), infliximab (I), adalimumab (A), certolizumab pegol (C), and golimumab (G), are FDA-approved biological agents for the treatment of rheumatoid arthritis and other chronic inflammatory conditions [8] (Figure 1). Naturally, pregnancy poses a challenge in treating chronic diseases in patients who plan to have children. The preservation of fertility and avoiding teratogenic or toxic effects must be considered during such therapies with anti-TNF-α agents. Romanowska-Próchnicka et al., 2021 [9] addressed these issues in detail.

Tubal factor accounts for approximately 30% of all cases of female fertility problems [10]. Sexually transmitted infections (STIs) caused by *Neisseria gonorrheae*, *Chlamydia trachomatis*, and *Mycoplasma genitalium* are significant, and are common risk factors for pelvic inflammatory disease, with subsequent obstruction of the fallopian tubes [11]. Moreover, pregnancy complications and adverse perinatal outcomes were more frequent in this group of patients. The prevalence of STIs is rising worldwide, and the rapid spread of antibiotic-resistant *N. gonorrheae* and *M. genitalium* necessitates the monitoring and development of new drugs and treatment regimens. The latest approaches to molecular diagnostics, treatments, and drug resistance in STIs are covered extensively by Smolarczyk et al., 2021 [12].

Preeclampsia (PE) is a common hypertensive disorder that affects first pregnancies. Defective uteroplacental vascular remodeling in PE results in the underdevelopment of low-resistance flow, with subsequent endothelial cell activation, intravascular inflammation, and syncytiotrophoblast stress [13]. Hayder et al., 2021 [14] add another brick to the constantly updated knowledge about the etiopathology of PE. They suggested that miR-210-3p overexpression in endovascular trophoblasts could impair spiral artery remodeling, thereby contributing to PE. The next paper also addresses PE pathophysiology. Magatti et al., 2021 [15] reviewed the role of B cells in immune-mediated conditions, including pregnancy, both as proinflammatory and regulatory cells. They indicate that disruption of the control mechanisms of perinatal cells for the maintenance of normal B-cell function may predispose patients to PE. Thus, B cells may be a potential target for perinatal cell-based therapy in PE.

It was suggested that epigenetic alterations of the placenta related to adipokines, and its delivery mode, are associated with obesity, Słabuszewska-Jóźwiak et al., 2021 [16] examined global placental DNA methylation status, as well as selected 5′-cytosine-phosphate-guanine-3′ (CpG) sites in adiponectin (ADIPOQ) and leptin (LEP) genes. Differences in the global methylation status in the placenta were observed depending on the delivery mode. In contrast, LEP and ADIPOQ demethylation indices did not significantly differ between the elective cesarean section and vaginal delivery groups.

Transplacental transfer of IgG and other immune factors during pregnancy provides passive immunity for the newborn and is crucial for protection against infection in early life. The impact of maternal microbiota and diet on maternal and cord-plasma concentrations, as well as the relationships among immunoglobulins, cytokines (CKs), and adipokines, was examined by Rio-Aige et al., 2021 [17]. They concluded that immune status during pregnancy, in terms of CKs and immunoglobulins, can influence the immune status of the infant at birth. Moreover, maternal dietary components (e.g., monounsaturated fatty acids, polyunsaturated fatty acids, and fiber) were positively associated with some immune factors (e.g., IgA) in cord samples. The composition of microbiota clustering also influenced the plasma profile of many CKs, some Ig, and adiponectin.

The fetal inflammatory response is a systemic activation of the major signaling pathways mediating nuclear factor kappa B (NF-kB) activation within the fetal immune system. This response increases the risk of infection-associated preterm birth (PTB) with increased neonatal morbidity and mortality. Na^+^/H^+^ exchanger regulatory factor-1 (NHERF1) is an adapter protein that can regulate intracellular signal transduction and, thus, influence NF-kB activation [18]. Kammala et al., 2020 [19] evaluated the levels of NHERF1 in human fetal membranes obtained after regular term deliveries and PTB. They also used an animal model of PTB induced using a lipopolysaccharide (LPS) and primary cultures of fetal membrane cells treated with LPS. After NHERF1 gene silencing using small interfering RNA, there was a significant reduction in NF-kB activation and IL-6. IL-8 production was demonstrated with increased levels of anti-inflammatory interleukin-10. The authors proposed that reducing NHERF1 expression could be a potential therapeutic strategy to reduce the risk of infection/inflammation associated with PTB.

The exact etiology of placental abruption—a relatively rare, yet severe, complication of pregnancy with partial or complete placental detachment before the birth of the fetus—is unknown [20]. A systematic review of the original papers on molecular changes in the maternal–fetal interface coexisting with placental abruption was carried out by Bączkowska et al., 2021 [21]. Consistently reported chronic noninfectious inflammation and excessive cytotoxic response within the placental tissue may indicate that the disruption of the immunological processes is an important etiological factor in this obstetrical complication.

Studies on placental immune responses to viruses have gained momentum, mainly due to the COVID-19 pandemic. Narang et al., 2021 [22] reviewed the general antiviral immunologic mechanisms utilized by trophoblast cells to protect the fetus, and discussed histopathologic evidence in cases where these mechanisms fail. Pregnant women are at higher risk of a severe course of severe acute respiratory syndrome 2 (SARS-CoV-2) infection. Bukowska-Ośko et al., 2021 [23] focused on the role of the mother–fetal–placenta interface in the protection and potential vertical transmission of SARS-CoV-2. The authors summarized the current knowledge on the antiviral activity of lactoferrin in pregnancy; additionally, they described, in detail, the expression of viral receptors and proteases, SARS-CoV-2 infection-related placental pathologies, and the presence of the virus in neonatal tissues and fluids.

## Figures and Tables

**Figure 1:**
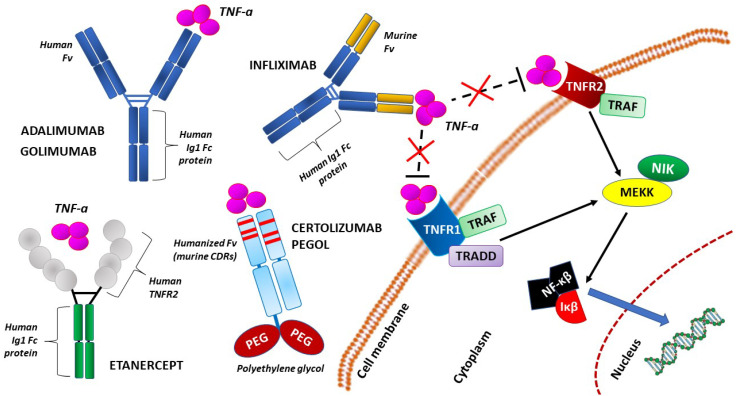
Simplified molecular structure of the representative compounds of tumor necrosis factor-alpha (TNF-α) inhibitors: infliximab, adalimumab, golimumab, certolizumab pegol, and etanercept. Adalimumab and golimumab both contain similar human IgG1 Fc and Fv components. Infliximab contains the variable regions of a mouse anti-TNF-alpha monoclonal antibody alongside its constant human IgG1 Fc. Etanercept contains a constant human IgG1 Fc region, as well as a TNFR2 region. Certolizumab pegol contains a PEGylated humanized Fab region. CDR—complementarity-determining region; Fab – antigen-binding fragment; Fc—fragment crystallizable; Fv—the smallest antibody fragment that contains a complete antigen-binding site; Iκβ—an enzyme complex IκB kinase; MEKK—mammalian mitogen-activated protein kinase kinase kinase; NF-κβ—nuclear factor kappa-light-chain-enhancer of activated B cells; NIK—NF-κB-inducing kinase; PEG—polyethylene glycol; TNF-α—tumor necrosis factor alpha; TNFR1—tumor necrosis factor receptor 1; TNFR2—tumor necrosis factor receptor 2; TRAF—tumor necrosis factor receptor-associated factor; TRADD—TNFR1-associated death domain protein.
